# A half blooded knot for wound closure

**DOI:** 10.1308/003588412X13171221591259e

**Published:** 2012-05

**Authors:** M Twyman

**Affiliations:** Epsom and St Helier University Hospitals NHS TrustUK

## BACKGROUND

We have been using the half blooded knot with a tuck for several years with no complications for closure of the extensor mechanism In elective knee replacements.

## TECHNIQUE

**Figure 1:**
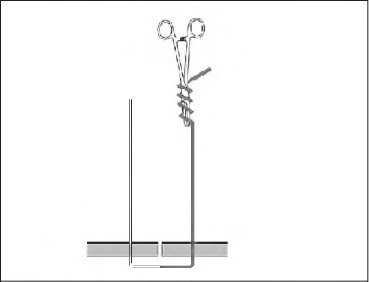
Wind the needle holders around the thread.

**Figure 2:**
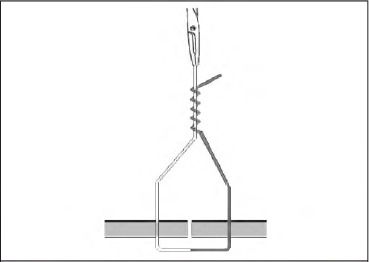
Grasp the working end (white) and pull It through the loops of the thread.

**Figure 3:**
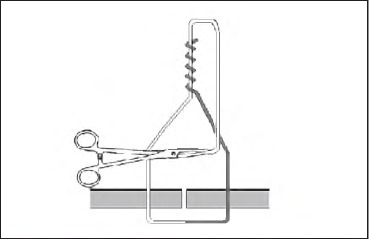
Pass the needle holders through the triangular loop

**Figure 4:**
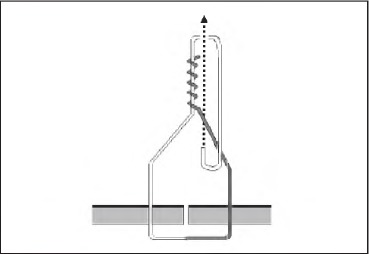
Withdraw the needle holders and re-grasp the working end as the standing end Is pulled tight

Tying a half blooded knot with a tuck is a four-stage technique. First, pass the suture through the tissue, which will give you two ends. Hold the standing end (grey, Fig 1) and use a wide circular motion with the needle holders facing the wound. Wind them four times around the thread in a similar manner to that a fencer would use with a sword. Grasp the working end (white, Fig 2) and pull It through the loops of the thread. Pinch the thread between thumb and forefinger where the two free ends converge. Still holding the working end, pass the needle holders through the triangular loop and then through the loop created (the tuck) (Fig 3). Release the working end and withdraw the needle holders. Re-grasp with no tension on this end as the standing end is pulled tight (Fig 4). The needle holders can then be used as a knot pusher If necessary.

## DISCUSSION

The half blooded knot with a tuck Is widely used in Ashing to attach the line to hooks. It has long since been known that a surgical knot is prone to slippage and weakens the suture material.^1^ However, as the half blooded knot will not slip in contrast to a surgical knot, the tall can be cut shorter, thereby reducing the total material left in the wound.
